# Effect of Laryngeal Mask Air Way Insertion versus Endotracheal Intubation over Hemodynamic Responses in Pediatrics Patient Who Underwent Ophthalmic Surgery at Menelik II Hospital, Addis Ababa: A Prospective Observational Study Design

**DOI:** 10.1155/2020/7021641

**Published:** 2020-06-01

**Authors:** Mohammed Suleiman Obsa, Azeb Lencha Sholla, Betelhem Girma Baraki, Getahun Dendir Welde, Temesgen Bati Gelgelu, Melese Meleku Kuruche

**Affiliations:** ^1^Wolaita Sodo University, Anesthesia Department, Wolaita Sodo, Ethiopia; ^2^Addis Ababa University, Anesthesia Department, Addis Ababa, Ethiopia; ^3^Wolaita Sodo University, School of Public Health, Wolaita Sodo, Ethiopia; ^4^Wolaita Sodo University, School of Nursing, Wolaita Sodo, Ethiopia

## Abstract

**Background:**

The airway of an anesthetized child is usually maintained with an endotracheal tube or laryngeal mask airway. However, both are related with some level of pressor response which may be risky in several groups of patient.

**Methods:**

An institutional-based prospective observational study design was employed. A systematic random sampling technique was used to select study participants. Data were entered into Epi info version 7 and transported to SPSS version 20 for analysis. Normality of the data was checked using Shapiro–Wilk tests. An independent *t* test was used to determine the mean differences between the two groups while the paired sample *t* test was used to determine the mean differences within the groups. A *p* value of less than 0.05 was used as a cut-off point for the presence of association.

**Results:**

The changes in systolic and diastolic blood pressure were returned to baseline values at five and three minutes in both groups, respectively. However, the changes in the heart rate and mean arterial pressure returned to baseline values in five minutes in the ETT group and three minutes in the LMA group. At baseline, the difference in systolic blood pressure between the two groups was not statistically significant (*p*=0.328).

**Conclusions:**

A significant hemodynamic pressor response was observed after the insertion of both LMA and ETT groups. However, the LMA group has less hemodynamic change as compared to the ETT group. Therefore, the practice of LMA insertion was strongly recommended.

## 1. Background

Laryngeal mask airway (LMA) and endotracheal tube (ETT) intubation are among the most important artificial airway devices used at the time of general anesthesia [1]. Traditionally, ETT insertion has been recognized as the foundation of maintaining adequate airway management. LMA offers a much less invasive way of maintaining airway as it does not pass through glottis. Both are noxious stimuli which elicit transient or marked sympathetic response [2].

The increased blood pressure and heart rate occurring due to reflex sympathetic discharge from response of laryngotracheal stimulation may have little consequences in healthy individuals, but may be more severe or even dangerous in patients with hypertension, myocardial insufficiency, and cardiovascular disease [3, 4]. In addition, the sudden rise in blood pressure can lead to left ventricular failure, cerebral hemorrhage, and myocardial ischaemia [5].

Many drugs and techniques have been used to attenuate pressor responses following intubation, but no single technique has gained universal acceptance. Use of LMA in place of ETT tube has been shown to have less hemodynamic response as its insertion requires neither visualization of cords nor the penetration of larynx [3]. Some studies found differences in hemodynamic changes [6–8], but other did not find any difference between LMA and ETT [6].

Many studies have shown the effect of different combinations of anesthetic drugs on reducing the side effect of tracheal intubation on patients' hemodynamic parameters [9, 10]. Despite of combinations of various anesthetic agents used, there was higher increase in hemodynamic changes in the ETT group than in the LMA group [11, 12]. However, a significant difference was not observed with propofol anesthesia [13]. Similarly, administering sevoflurane and remifentanil combination did not find significant difference in hemodynamic changes [14].

In this study, we compared hemodynamic changes after LMA insertion and endotracheal ETT intubation to find out a better way of controlling the airway.

## 2. Methods and Materials

### 2.1. Study Setting

An institutional-based prospective observational study design was carried out at Menelik II Hospital from February 30 to April 30, 2017.

### 2.2. Study Participants

All pediatric patients who underwent elective ophthalmic surgery at Menelik II referral hospital were source population while all selected elective pediatric patients who underwent ophthalmic surgery with ETT or LMA at Menelik II hospital from February 30 to April 30, 2017 were study populations. In this study, ASA I and ASA II pediatrics patients aged between 1–12 years, patients with anticipated difficult air way, patients who underwent only mask ventilation, and parents who are unwilling to give written consent were excluded.

#### 2.2.1. Sample Size

Continuous outcomes formula was used to calculate the sample size based on a previous study done in India [15], which showed a heart rate mean and standard deviation of 121.16 ± 19.90 and 111.24 ± 9.20 among the endotracheal intubation and laryngeal mask airway groups, respectively.

(1) $$\begin{array}{c} n=\left(S_{1}^{2}+S_{2}^{2}\right){\left(Z\frac{\alpha }{2}+Z\beta \right)}^{2},\\ {\left(\mu_{1}-\mu_{2}\right)}^{2}, \end{array}$$

where *Z α*/2 = 1.96 for a *p*=0.05 (95% confidence interval), *Zβ* = 0.84 for 20% beta error, *S* = standard deviation, and *μ* = heart rate mean.

(2) $$\begin{array}{c} n={\left(19.90\right)}^{2}+{\left(9.20\right)}^{2}{\left(1.96+0.84\right)}^{2}{\left(121.16-111.24\right)}^{2},\\ n=38\;\mathrm{patients}\;in\;\mathrm{each}\text{ group}. \end{array}$$

Sample size was increased by 10% in order to replace for any dropouts so that the total sample size (*n*) became 42 in each group.

#### 2.2.2. Sampling Procedure

A systematic random sampling technique was used to select study participants. The mid-year population from situational analysis was 1080, and the size of population in 2 months was 360. Accordingly, the calculated sampling interval *K*_th_ _=_ *N*/*n* = 360/84 = 4 and then, a random number 2 was chosen by a lottery method and every 4th subsequent patients on the day of data collection were included. This process was continued till the required sample size was achieved.

### 2.3. Data Collection Tools and Procedures

Structured questionnaires were used to gather information from patient's charts and to interview patient's family. Two Bachelor of Science (BSC) holder's and one Master of Science (MSC) holder's anesthetists with at least four year work experience were involved as data collectors and supervisors, respectively. Preoperative vital signs were considered as baseline line vital signs. Monitoring included electrocardiography, heart rate, pulse oximetry, end-tidal carbon dioxide measurement, and noninvasive blood pressure were applied, and an intravenous (IV) access was secured. Jaw thrust with mask ventilation was used for 3 min with 2% halothane in oxygen. All patients were induced by 2.5 mg/kg propofol, 1 mg/kg of ketamine, and 0.8 mg/kg pethidine followed by induction of anesthesia with 1 mg/kg of suxamethonium. After ensuring adequacy of ventilation, vecuronium bromide 0.1 mg/kg was given for neuromuscular (NM) blockade. Surgery was started after five minutes of insertion of airway devices. Anesthesia was maintained using halothane in oxygen and vecuronium top ups for NM blockade. At the end of the surgery, the residual NM blockade was antagonized with neostigmine and atropine in appropriate dosages.

#### 2.3.1. Data Quality Assurance

A pretest was done on 5% of the sample size outside study area. Training was given for data collectors and supervisors. Investigator made regular supervision and follow-up.

#### 2.3.2. Data Management and Analyzing Procedure

Data were entered into Epi info version 7 computer programs and exported to SPSS version 20 computer programs for further analysis and cleaning. Before further analysis, normality of the data was checked using Shapiro–Wilk tests. An independent sample *t* test and a paired sample *t* test were used to determine the mean differences between the two groups and within the groups, respectively. In the meantime a *p* value < 0.05 was used to determine the presence of association.

### 2.4. Operational Definitions

  Hemodynamic changes: changes in vital signs to airway manipulation.
  Vital Signs: in this study it included BP, MAP, and PR.
  Easy insertion: insertion of ETT or LMA in one attempt.
  Difficult insertion: insertion of ETT or LMA more than two attempts.

## 3. Result

### 3.1. Socio-Demographic Characteristics

A total of 84 pediatrics ophthalmic patients were enrolled in the study of which the mean age of respondents for LMA was 6.45 ± SD (3.217) while the mean age of respondents for the ETT group was 6.57 ± SD (3.013). The overall mean age of respondents was 7.10 ± SD (6.033) (minimum 1 and maximum 12). It was also found that the overall mean weight of patients was 23.38 ± SD (15.803) (minimum 10 and maximum 32). 95.23% and 92.85% of patients were intubated with one attempt of LMA and ETT insertion, respectively (Table 1).

### 3.2. Mean Heart Rate of Pediatric Patients

The mean changes in the heart rate following ETT intubation and LMA insertion were 29.63 and 15.48, respectively. The elevation in the heart rate significantly persisted for a longer period of time in the ETT group. These elevations were returned to the baseline value in 5 minutes in the ETT group and 3 minutes in the LMA group. By 5 minutes, there was no significant difference between the two groups (*p* value = 0.627) (Table 2).

### 3.3. Mean Systolic Blood Pressure of Pediatric Patients

At baseline, the difference in systolic blood pressure (SBP) between the two groups was not statistically significant (*p*=0.328). The increase within and between the two groups was statistically significant after insertion at (*p* < 0.05). It took 5 minutes for both ETT and LMA values to return to baseline (Table 3).

### 3.4. Mean Diastolic Blood Pressure of Pediatric Patients

The baseline diastolic blood pressure was comparable between the two groups. After insertion, both groups showed an increase in diastolic blood pressure (DBP) that was statistically significant within and between the groups. At three minutes, the difference within the LMA group was not statistically significant (*p* value = 0.143) while the difference within the ETT group was statistically significant (*p* value = 0.003) (Table 4).

### 3.5. Mean Arterial Blood Pressure of Pediatric Patients

There is no significant difference in the baseline mean arterial pressure (MAP) between the two groups. After instrumentation, the ETT group had an increase in MAP that was higher and more persistent as compared to the LMA group (Table 5).

## 4. Discussion

Pediatric ophthalmic surgery usually requires general anesthesia and tracheal intubation that may have deleterious effects on cardiovascular function [16, 17]. LMA has been found to be superior to tracheal intubation in terms of maintaining stable vital signs [18] but positive pressure ventilation could become a challenge in certain cases. LMA offers the advantage of providing a better seal in the oropharynx to allow ventilation at much higher pressure and to protect the stomach from gastric insufflation [19]. In our country Ethiopia, pressor response to insertion of supraglottic devices have not been compared to tracheal intubation and changes in vital signs following insertion have not been evaluated.

According to the result of the present study, there was a variation in hemodynamic parameters after the insertion of both LMA and ETT. Similarly, another study also found that a significant increase in mean value was observed among the ETT group [20, 21]. The findings of our study were also closely correlated with another multiple clinical studies as hemodynamic changes were less during the LMA placement than during tracheal intubation [22–24]. This may be likely due to insertion of an LMA is easier and takes a shorter time as compared to the ETT insertion so that the degree of stimulating sympathetic nervous system was slower and shorter.

According to the result of the present study, the LMA group showed a significant increase in HR and SBP, following insertion. However, the changes in the LMA group were significantly lower compared to the ETT group which agrees with other study [25]. This may be because LMA did not go through the trachea subsequently that caused less stimulation of the supraglottic region causing less activation of the hemodynamic response reflex. This explanation was supported by another study [26, 27].

Similar to our study, several other studies have demonstrated that the hemodynamic response to LMA is short lived as compared to that of ETT [23, 27]. The results of this study are also similar to those of the study done in Nigeria and Austria [20, 28]. The greater and more persistent changes in cardiovascular parameters seen with the ETT group is probably due to the sympthatoadrenal response caused by stimulation of the supraglottic region and that of the trachea [23]. This may also be likely due to insertion of laryngoscope with ETT may take longer time to perform which could translate to a longer stimulation period, leading to a greater hemodynamic response [29].

The limitations of this study were a large-scale prospective study that could not be conducted because of the time constraint and double blinding which was not possible due to operation theatre setup. This could mean that an element of observation bias was not completely removed from the study.

## 5. Conclusions

In this study, the magnitude and extent of increase in the heart rate, systolic blood pressure, and mean arterial pressure were significantly higher after endotracheal intubation than LMA insertion. Therefore, anesthetists should commonly use LMA for providing adequate airway management and reserve ETT for those who are contraindicated to use LMA. Further studies should be conducted on larger populations using a comparative randomized clinical trial to get a bigger picture of how the effect of the LMA and ETT would be in the Ethiopian population.

## Figures and Tables

**Table 1:** Socio-demographic characteristics of study subjects.

Group	Number	Age in years mean ± SD	Weight (kgs) mean ± SD	Sex		ASA	
				Male	Female	ASA I	ASA II
LMA	42	6.45 ± 3.217	20.90 ± 6.435	20 (47.6%)	22 (52.4%)	38 (90.5%)	4 (9.5%)
ETT	42	6.57 ± 3.013	23.48 ± 15.810	21 (50%)	21 (50%)	40 (95.2%)	2 (4.8%)

**Table 2:** The mean heart rate among ETT and LMA of study subjects.

Heart rate	ETT mean ± SD	*p* value for difference within ETT group	LMA Mean ± SD	*p* value for difference within LMA group	*p* value for difference between ETT and LMA groups
Baseline HR	113.55 ± 25.885	—	116.86 ± 26.276	—	0.546
After insertion	143.18 ± 26.368	0.000	132.34 ± 26.453	0.000	0.003
At 1 min.	142.24 ± 25.236	0.000	130.78 ± 25.342	0.000	0.001
At 3 min.	139.24 ± 25.324	0.000	115.64 ± 22.158	0.156	0.316
At 5 min.	112.86 ± 24.467	0. 328	110.43 ± 18.948	1.238	0.627

**Table 3:** Mean systolic blood pressure among ETT and LMA of study subjects.

Systolic blood pressure	ETT mean ± SD	*p* value for difference within ETT group	LMA Mean ± SD	*p* value for difference within LMA group	*p* value for difference between ETT and LMA groups
Baseline SBP	109.67 ± 19.034	—	110.95 ± 13.458	—	0.328
After insertion	132.58 ± 23.863	0.000	122.42 ± 14.165	0.000	0.004
At 1 min.	131.68 ± 24.376	0.000	122.85 ± 13.254	0.002	0.336
At 3 min.	125.45 ± 22.862	0.468	115.12 ± 12.247	0.078	0.439
At 5 min.	109.15 ± 17.186	0.124	109.64 ± 12.125	0.146	0.303

**Table 4:** Mean diastolic blood pressure among ETT and LMA of study subjects.

Diastolic blood pressure	ETT mean ± SD	*p* value for difference within ETT group	LMA Mean ± SD	*p* value difference within LMA group	*p* value for difference between ETT and LMA groups
Baseline DBP	73.45 ± 14.492	—	72.31 + 11.104	—	0.532
After insertion	84.32 ± 15.860	0.000	78.16 + 15.134	0.000	0.002
At 1 min.	85.27 ± 16.103	0.000	74.28 + 14.176	0.000	0.213
At 3 min.	73.85 ± 13.628	0.003	72.25 + 10.173	0.143	0.219
At 5 min.	70.44 ± 12.182	0.274	69.35 + 10.114	0.154	0.623

**Table 5:** Mean arterial blood pressure among ETT and LMA of study subjects.

MAP	ETT mean ± SD	*p* value for difference within ETT group	LMA Mean ± SD	*p* value for difference within LMA group	*p* value for difference between ETT and LMA groups
Baseline MAP	91.79 ± 14.851	—	86.38 ± 12.253	—	0.015
After insertion	115.14 ± 17.213	0.000	106.64 ± 18.135	0.000	0.001
At 1 min.	112.34 ± 16.947	0.000	100.84 ± 18.214	0.006	0.416
At 3 min.	98.48 ± 16.153	0.007	85.78 ± 10.378	0.035	0.115
At 5 min.	89.58 ± 14.143	0.454	84.14 ± 10.211	0.113	0.237

## Data Availability

The data used to support the findings of this study are available from the corresponding author upon request.

## Abbreviations

AAU: Addis Ababa University
LMA: Laryngeal mask airway
ETT: Endotracheal tube.
